# Changes in vitamin D status among adults from the COVID-19 pandemic to post-pandemic normality

**DOI:** 10.3389/fnut.2024.1407890

**Published:** 2024-08-02

**Authors:** Yanzhao Chen, Guilian Kong

**Affiliations:** Demonstration Laboratory of Quality Control for in vitro Analysis, Department of Nuclear Medicine, Henan Provincial People's Hospital, Zhengzhou University People's Hospital, The Clinical Medical College of Provincial Hospital of Zhengzhou University, Zhengzhou, Henan, China

**Keywords:** vitamin D, adults, COVID-19, pandemic, China

## Abstract

**Introduction:**

The COVID-19 pandemic has prompted widespread lockdown measures globally, significantly impacting daily activities and outdoor exposure. This study investigates the effect of the pandemic on vitamin D levels and the prevalence of vitamin D deficiency in the adult population, with a focus on gender-specific differences.

**Methods:**

A total of 1525 adults from Henan Provincial People’s Hospital were included. Serum 25-hydroxyvitamin D (25(OH)D) levels were measured using the Elecsys® Vitamin D total kit on the Roche Cobas® 8000 analyzer. The Clinical Application Consensus on Vitamin D and Its Analogs defined deficiency as 25(OH)D levels below 20 ng/ml. Statistical analysis was performed using SPSS 23.0 and GraphPad Prism 8 software.

**Results:**

The overall 25(OH)D levels increased from 18.14 ng/ml [IQR: 13.78, 23.68] in 2022 to 19.15 ng/ml [IQR: 14.88, 25.01] in 2023 (*p*=0.004). Males exhibited significant improvement in 25(OH)D levels from 18.01 ng/ml [IQR: 14.10, 23.53] in 2022 to 20.49 ng/ml [IQR: 16.11, 26.01] in 2023 (*p*<0.001). The prevalence of vitamin D deficiency decreased from 62% in 2022 to 54.9% in 2023 (*p*=0.009), with a notable reduction in males (64.1% in 2022 to 47.2% in 2023). Among 168 individuals tested in both years, 25(OH)D levels increased from 20.73 ± 9.37 ng/ml in 2022 to 22.28 ± 8.59 ng/ml in 2023 (*p*=0.012), and the deficiency rate decreased from 58.3% in 2022 to 47.0% in 2023 (*p*=0.038). The 40–49 age group showed significant improvement in 25(OH)D levels from 16.10 ng/ml [IQR: 12.41, 21.18] in 2022 to 18.28 ng/ml [IQR: 13.91, 23.86] in 2023 (*p*=0.005), with a reduction in deficiency rate from 72.8% to 59.9% (*p*=0.02). Furthermore, in February, March, and April, 2022, 25(OH)D levels were significantly lower compared to 2023 (*p*<0.001, *p*=0.002, *p*<0.001, respectively), accompanied by a higher prevalence of vitamin D deficiency (*p*<0.001, *p*=0.015, *p*<0.001, respectively).

**Discussion:**

This study demonstrates that the COVID-19 pandemic significantly impacted vitamin D levels, leading to an increased prevalence of deficiency, particularly among males. These findings highlight the critical importance of maintaining sufficient outdoor activities to ensure adequate vitamin D levels. The data underscore the need for public health strategies to address potential deficiencies during prolonged periods of limited outdoor exposure.

## 1 Introduction

Since the emergence of the COVID-19 pandemic in Wuhan, China, in early 2020, China has implemented stringent measures to control the spread of the virus (1). These measures entail mandatory mask usage, strict enforcement of social distancing, and stay-at-home orders when necessary. While some regions in China adopted a “zero-COVID” policy starting from August 2021, the city of Zhengzhou in Henan Province enforced rigorous lockdown measures during specific periods: January 5 to February 9, May 3 to May 17, and October 14 to December 14, 2022. These measures significantly curtailed outdoor activities, potentially leading to diminished levels of vitamin D, which is primarily synthesized in the skin through exposure to sunlight, specifically ultraviolet B radiation (2).

Vitamin D serves as a prohormone for a lipid-soluble steroid hormone. In its native form, vitamin D does not exhibit biological activity and necessitates hepatic conversion to its principal storage form, 25(OH)D (3). Following this, it undergoes intracellular transformation within vitamin D receptor-expressing cells, culminating in the generation of the biologically active metabolite, 1,25-dihydroxyvitamin D (4). Vitamin D plays a critical role in mediating calcium absorption and modulating bone metabolism within the human body (5). Moreover, vitamin D deficiency has been linked to unfavorable outcomes in COVID-19 patients, as well as increased susceptibility to bacterial and viral infections (6–8). Some studies have identified a strong correlation between low vitamin D levels and worse COVID-19 outcomes, independent of initial disease severity. This excludes reverse causality and supports vitamin D as an independent predictor of disease progression (9). Furthermore, emerging data have provided intriguing insights into the association between vitamin D levels and Long COVID occurrence. Long COVID, characterized by prolonged symptoms following the acute phase of infection, has been associated with various risk factors, including low vitamin D levels (10). This expands the potential therapeutic applications of vitamin D in managing COVID-19 and its associated comorbidities.

Vitamin D exerts influence on the expression of over 1,000 genes and is implicated in various conditions, including diabetes, diverse cancer types, cardiovascular diseases, autoimmune disorders, and congenital immune disorders (6, 11–13). There is also growing evidence linking vitamin D levels with the response to anti-SARS-CoV-2 vaccination (14). Vitamin D, known for its immunomodulatory effects, may influence the efficacy of vaccines, making it a critical factor to consider in vaccination strategies. Moreover, the non-skeletal effects of vitamin D have been the subject of recent international consensus, which emphasizes its role beyond bone health (15). Of particular interest is the association between vitamin D and diabetes, where recent findings suggest a beneficial effect of adequate vitamin D levels in reducing the risk of diabetes (16).

Previous studies have yielded conflicting findings regarding the impact of the COVID-19 pandemic on vitamin D levels and deficiency rates. Some studies have reported no effect or even an elevation in vitamin D levels and a reduction in deficiency rates among populations (17–19). Conversely, other studies have observed a decline in vitamin D levels and an increase in deficiency rates (20). Currently, there is a dearth of research specifically investigating the influence of the COVID-19 pandemic on vitamin D levels in the adult population of China. Therefore, the primary objective of this study is to perform a comprehensive statistical analysis and comparative assessment of vitamin D levels among a population of healthy adults residing in Zhengzhou, Henan Province. Specifically, we aim to investigate potential variations in vitamin D levels and the prevalence of vitamin D deficiency between two distinct time periods: the COVID-19 pandemic in 2022 and the subsequent post-pandemic phase in 2023. The overarching aim is to elucidate the potential impact of the COVID-19 epidemic on vitamin D levels and the associated deficiency rates.

## 2 Methods

### 2.1 Study population

A cohort of 1,525 individuals who underwent comprehensive health examinations at the Henan Provincial People’s Hospital Examination Center between January 2022 and December 2023 was selected as the study population. The cohorts in 2022 and 2023 are distinct groups, with 2022 representing data during the pandemic and 2023 representing post-pandemic data. The cohorts consisted of 776 males and 749 females. The inclusion criteria for the study population were as follows: (1) age 18 years or older, and (2) absence of known medical conditions associated with abnormal levels of 25(OH)D, such as hyperparathyroidism or hypoparathyroidism, hyperthyroidism, severe liver or kidney diseases, post-gastrectomy status, severe infection, malignant tumors, among others. The study protocol adhered to the principles outlined in the Helsinki Declaration and received approval from the Hospital Ethics Review Committee. All procedures were conducted as part of routine clinical practice. Informed consent was waived due to the retrospective nature of the study.

### 2.2 Data collection and 25(OH)D measurement

Demographic information, including name, gender, age, identification number, examination date, and relevant clinical data such as underlying diseases, were obtained and extracted from the hospital’s electronic system. Serum 25(OH)D levels were measured using the Elecsys^®^ Vitamin D total kit (Roche Diagnostics) on the Roche Cobas^®^ 8,000 modular analyzer, specifically the e602 analyzer. The cutoff for defining vitamin D deficiency was based on the Clinical Application Consensus on Vitamin D and Its Analogs published by the Chinese Society of Osteoporosis and Bone Mineral Research in February 2018, which considered serum 25(OH)D levels below 20 ng/mL as indicative of deficiency.

### 2.3 Statistical analysis

Statistical analysis was performed using SPSS 23.0 software, and data visualization was carried out using GraphPad Prism 8 software. Non-normally distributed continuous variables were described using the median with interquartile range (IQR) and analyzed using nonparametric tests. Paired t-tests were utilized for individuals who underwent 25(OH)D testing in both years, with continuous variables presented as mean ± standard deviation. Categorical variables were analyzed using chi-square tests. Statistical significance was set at a two-tailed *p*-value less than 0.05.

## 3 Results

### 3.1 Distribution characteristics of the study population

A total of 1,525 healthy adult participants were enrolled from the Physical Examination Center of Henan Provincial People’s Hospital for this study. The demographic characteristics and distribution of the study population are summarized in Table 1. In 2022, during the period of epidemic control, there were 479 participants (287 males, 192 females). In 2023, following the complete relaxation of epidemic measures, there were 1,046 participants (489 males, 557 females). Among the 1,525 participants, a subset of 168 individuals (101 males, 67 females) underwent continuous 25(OH)D testing for both years (2022 and 2023). Notably, there was a statistically significant difference in gender distribution between these 2 years. The collected data was stratified by age groups: 18–29 years, 30–39 years, 40–49 years, 50–59 years, and ≥ 60 years. There was no statistically significant difference in the age distribution between the populations of 2022 and 2023. However, there was a statistically significant difference in serum 25(OH)D levels and the prevalence of vitamin D deficiency between the years 2022 and 2023.

**Table 1:** Characteristics of study subjects in the year 2022 (during COVID-2019 pandemic) compared to 2023 (post COVID-2019 pandemic).

Variables	2022(*n* = 479)	2023(*n* = 1,046)	*p*-value
Gender, n (%)			
Male	287(59.9%)	489(46.7%)	<0.001
Female	192(40.1%)	557(53.3%)	
Age (years)	45(36, 56)	47(35, 56)	0.429
Age (years), n (%)			
18–29	36(7.5%)	103(9.8%)	0.051
30–39	148(30.9%)	273(26.1%)	
40–49	114(23.8%)	212(20.3%)	
50–59	108(22.5%)	272(26.0%)	
≥60	73(15.2%)	186(17.8%)	
25(OH)D (ng/mL)	18.14(13.78, 23.68)	19.15(14.88, 25.01)	0.004
VD status (ng/mL), n (%)			
<20	297(62%)	574(54.9%)	0.009
≥20	182(38%)	472(45.1%)	

COVID-19, coronavirus disease 2019; 25(OH)D, 25-hydroxy vitamin D.

### 3.2 Impact of the COVID-19 pandemic on vitamin D levels in the population

Serum levels of 25(OH)D were assessed using median and quartile values in the study participants. Statistical analysis revealed a significant difference in 25(OH)D levels between the year of during COVID-19 pandemic (2022) and the year of post COVID-19 pandemic (2023), with a *p*-value of 0.004. Within the same gender group, males exhibited significantly lower serum levels of 25(OH)D in 2022 compared to 2023 (*p* < 0.001). However, no statistically significant difference was observed in 25(OH)D levels among females (*p* = 0.908). Further analysis indicated that there was no statistically significant disparity in vitamin D levels between males and females in 2022 (*p* = 0.774). Nonetheless, in 2023, the vitamin D levels among males exhibited a marked increase in comparison to females, with a statistically significant difference (*p* < 0.001) as indicated in Table 2.

**Table 2:** The 25(OH)D concentration and the rate of vitamin D deficiency in different gender groups in 2022(during COVID-2019 pandemic) compared to 2023 (post COVID-2019 pandemic).

Variables	2022(*n* = 479)	2023(*n* = 1,046)	*p*-value
Total M(P25-P75)	18.14(13.78, 23.68)	19.15(14.88, 25.01)	0.004
Male M(P25-P75)	18.01(14.10, 23.53)	20.49(16.11, 26.01)	<0.001
Female M(P25-P75)	18.28(13.27, 24.39)	17.69(13.72, 23.43)	0.908
*p*-value	0.774	<0.001	
VD status <20 (ng/mL), n (%)			
Total	297(62%)	574(54.9%)	0.009
Male	184(64.1%)	231(47.2%)	<0.001
Female	113(58.9%)	343(61.6%)	0.505
*p*-value	0.245	<0.001	

The concentration unit of 25(OH)D was ng/mL. COVID-19, coronavirus disease 2019; 25(OH)D, 25-hydroxy vitamin D.

Among different age groups, individuals aged 40–49 years demonstrated significantly lower serum levels of 25(OH)D in 2022 compared to 2023 (*p* = 0.005), Subsequent analysis demonstrated a statistically significant elevation in vitamin D levels among individuals aged 50–59 years and those above 60 years during both the years 2022 and 2023, in contrast to the remaining age cohorts (*p* < 0.001). The detailed results are presented in Table 3.

**Table 3:** The 25(OH)D concentration and the rate of vitamin D deficiency in different age groups in 2022(during COVID-2019 pandemic) compared to 2023 (post COVID-2019 pandemic).

Age (years)	2022		2023		*p*-value	2022	2023	*p*-value
	n	M (P25, P75)	n	M (P25, P75)		25(OH)D < 20 ng/mL, n (%)		
18–29	36	16.09(13.43,21.41)	103	15.08(12.81,21.26)	0.706	26(72.2%)	75(72.8%)	0.945
30–39	148	17.10(12.87,20.49)	273	17.59(13.84,22.12)	0.059	110(74.3%)	179(65.6%)	0.064
40–49	114	16.10(12.41,21.18)	212	18.28(13.91,23.86)	0.005	83(72.8%)	127(59.9%)	0.020
50–59	108	20.14(15.31,27.13)	272	21.63(16.34,27.76)	0.322	52(48.1%)	121(44.5%)	0.518
>60	73	24.20(17.16,33.70)	186	21.47(17.18,29.88)	0.200	26(35.6%)	72(38.7%)	0.644
*p*		<0.001		<0.001		<0.001	<0.001	

COVID-19, coronavirus disease 2019; 25(OH)D, 25-hydroxy vitamin D.

Furthermore, the population was stratified by month, and a comparative analysis of serum 25(OH)D levels between the 2 years was conducted. The statistical analysis revealed that the levels of 25(OH)D in February, March, and April of 2022 were significantly lower compared to the corresponding period in 2023, with *p*-values of <0.001, 0.002, and < 0.001, respectively. In contrast, the testing results in October 2022 were higher than in 2023, with a statistically significant difference (*p* = 0.026). However, there were no statistically significant differences observed in the remaining months when comparing the results between 2022 and 2023. The detailed results are presented in Table 4.

**Table 4:** The 25(OH)D serum concentration and the rate of vitamin D deficiency in each month of the year 2022 (during COVID-2019 pandemic) and 2023 (post COVID-2019 pandemic).

	2022		2023		*p*-value	2022	2023	*p*-value
	n	M (P25, P75)	n	M (P25, P75)		25(OH)D < 20 ng/mL, n (%)		
Jan	13	15.06(12.23, 18.66)	57	15.73(12.69, 21.49)	0.345	10(76.9%)	42(73.7%)	0.809
Feb	56	12.23(10.46, 14.81)	50	17.24(13.70, 23.84)	<0.001	54(96.4%)	31(62.0%)	<0.001
Mar	38	16.54(11.97, 21.90)	93	19.86(16.40, 25.28)	0.002	28(73.7%)	47(50.5%)	0.015
Apr	76	15.91(12.53, 19.49)	130	20.16(15.45, 25.46)	<0.001	61(80.3%)	70(53.8%)	<0.001
May	63	18.40(13.96, 21.93)	123	19.02(15.02, 23.85)	0.297	41(65.1%)	68(55.3%)	0.199
June	50	23.58(17.29, 29.91)	98	20.16(16.64, 27.92)	0.090	18(36.0%)	48(49.0%)	0.133
July	46	22.42(16.72, 26.60)	81	22.55(18.14, 29.36)	0.237	18(39.1%)	26(32.1%)	0.423
Aug	42	22.09(17.56, 31.75)	52	23.89(17.62, 30.26)	0.957	16(38.1%)	22(42.3%)	0.679
Sep	55	19.45(14.47, 23.70)	65	22.27(16.33, 26.86)	0.196	30(54.5%)	25(38.5%)	0.078
Oct	27	19.89(15.35, 30.50)	100	17.01(13.88, 21.64)	0.026	14(51.9%)	66(66.0%)	0.177
Nov	8	17.58(13.38, 23.30)	97	16.40(13.21, 21.77)	0.704	5(62.5%)	68(70.1%)	0.653
Dec	5	35.07(13.87, 41.58)	100	16.33(12.42, 21.16)	0.078	2(40.0%)	67(67.0%)	0.215
*p*		<0.001		<0.001		<0.001	<0.001	

The concentration unit of 25(OH)D was ng/mL. COVID-19, coronavirus disease 2019; 25(OH)D, 25-hydroxy vitamin D.

**Figure 1:**
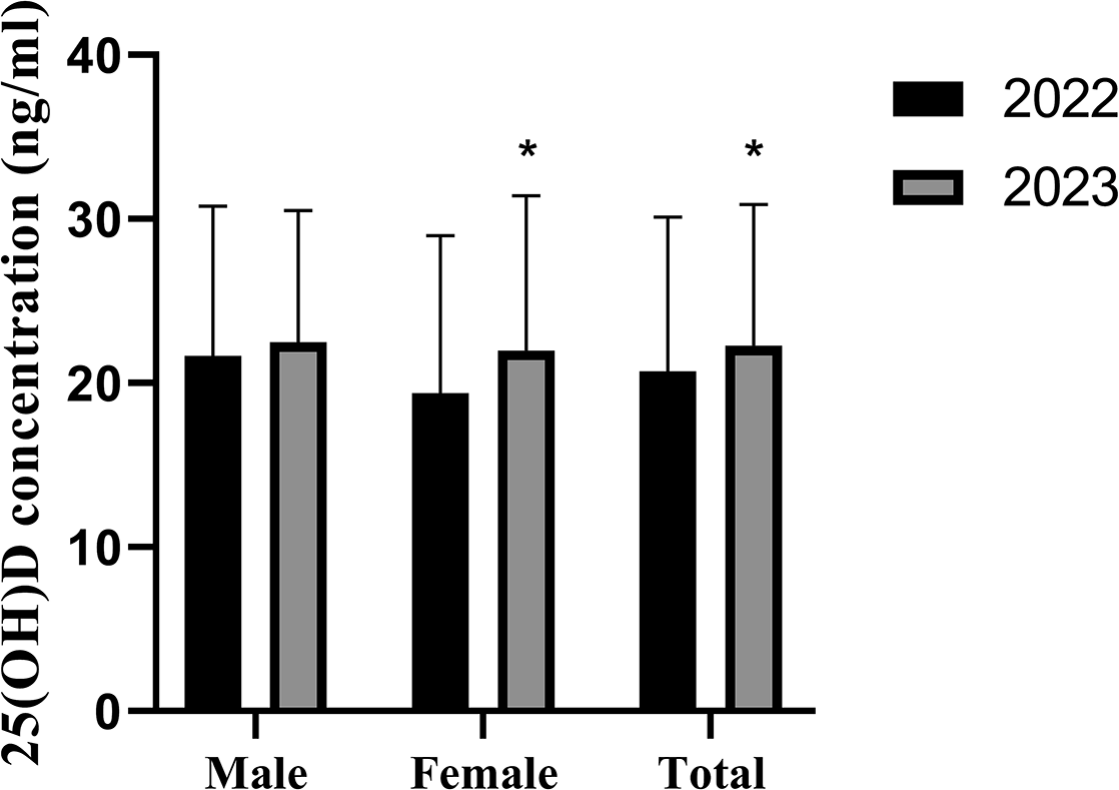
The 25(OH)D was tested in 168 participants (male, 101 and female, 67) both of the year 2022 and 2023. Error bars indicated mean ± SD, *p < 0.05.

For the subset of 168 individuals who underwent continuous 25(OH)D testing over two consecutive years, the results were reported as mean ± standard deviation. The overall testing results in 2022 (20.73 ± 9.37) were significantly lower than those in 2023 (22.28 ± 8.59), with a *p*-value of 0.012. Among females, the testing results in 2022 (19.37 ± 9.60) were significantly lower than those in 2023 (21.97 ± 9.46), with a p-value of 0.015. However, among males, there was no statistically significant difference in the testing results between 2022 (21.64 ± 9.15) and 2023 (22.49 ± 8.01), with a p-value of 0.253, as presented in Figure 1.

### 3.3 Impact of the COVID-19 pandemic on the prevalence of vitamin D deficiency in the population

In the year during COVID-19 pandemic in 2022, a total of 297 individuals (62% of the total population) had serum 25(OH)D levels below 20 ng/mL. In 2023, the number of individuals with vitamin D deficiency increased to 574, representing 54.9% of the total population. Statistical analysis revealed a significant difference in the prevalence of vitamin D deficiency between 2022 and 2023 (*p* = 0.009). When stratifying by gender, the prevalence of vitamin D deficiency among males was 64.1% in 2022, which was higher than the 47.2% in 2023, with a statistically significant difference (*p* < 0.001). However, no statistically significant difference was observed in the prevalence of vitamin D deficiency among females (*p* = 0.505). Further analysis demonstrated that there was no statistically significant difference in the prevalence of vitamin D deficiency between males and females in 2022 (*p* = 0.245). However, in 2023, the prevalence of vitamin D deficiency among males was significantly lower in comparison to females, with a statistically significant difference (*p* < 0.001). The results are presented in Table 2.

When comparing by age group, the prevalence of vitamin D deficiency in the 40–49 age group in 2022 was 72.8%, higher than the 59.9% in 2023, with statistically significant differences (*p* = 0.020). No statistically significant difference was observed in the prevalence of vitamin D deficiency in other age groups. Further analysis revealed a significantly lower prevalence of vitamin D deficiency among individuals aged 50–59 years and those above 60 years in both 2022 and 2023, as compared to other age groups. This difference was statistically significant (*p* < 0.001). The results are presented in Table 3.

**Figure 2:**
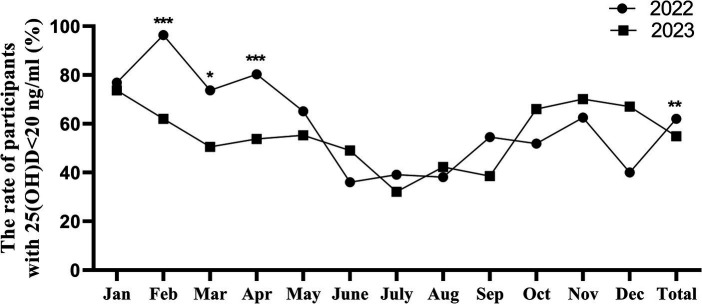
The monthly vitamin D deficiency rates for 2022 and 2023, *p < 0.05, **p < 0.01, ***p < 0.001.

When comparing by month, the prevalence of vitamin D deficiency in February, March, and April of 2022 was 96.4, 73.7, and 80.3%, respectively, higher than the corresponding periods in 2023, which were 62.0, 50.5, and 53.8%, respectively, with statistically significant differences (*p* < 0.001, 0.015, and < 0.001, respectively). No statistically significant difference was observed in the prevalence of vitamin D deficiency in other months. The results are presented in Table 4 and Figure 2.

**Figure 3:**
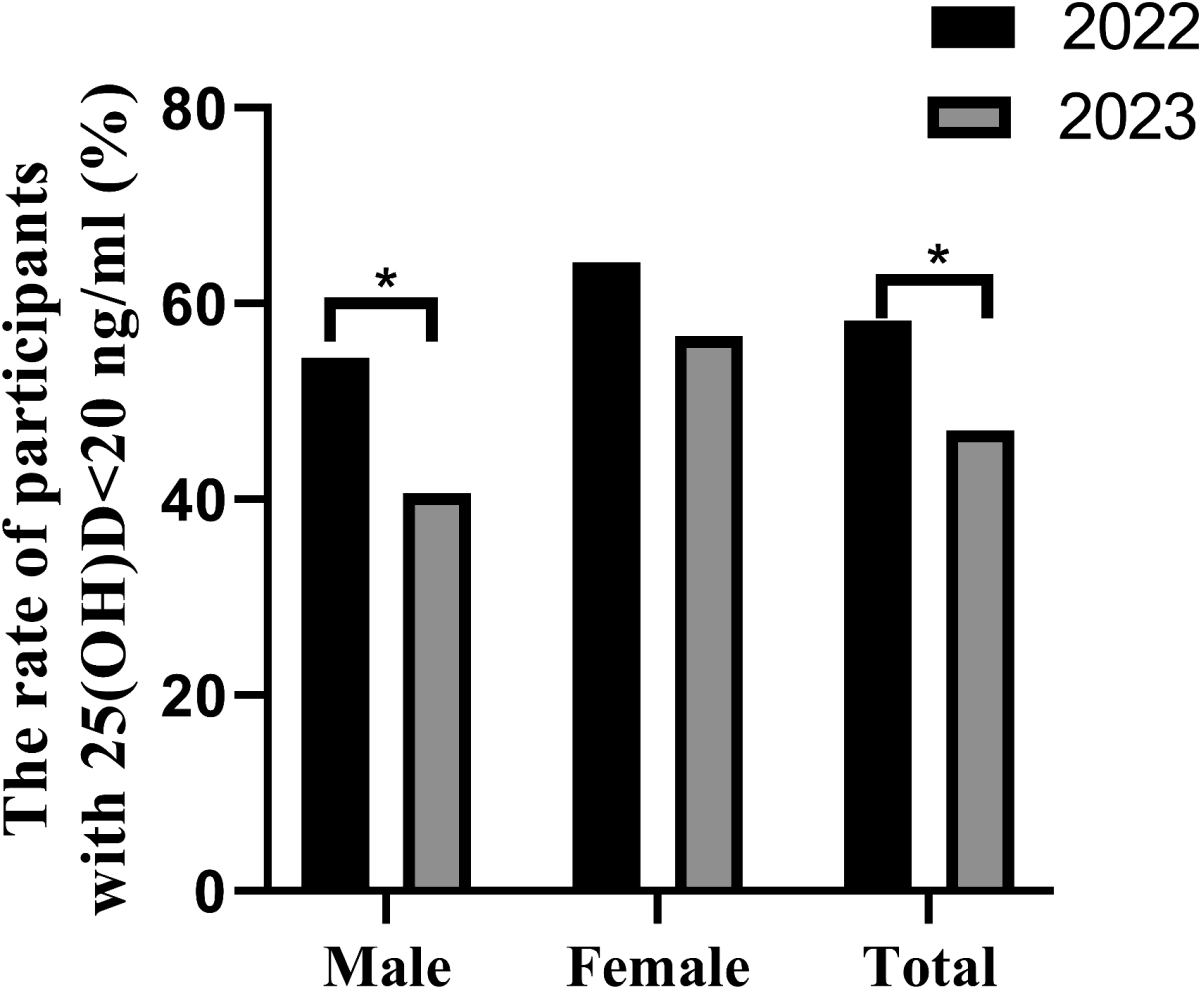
The rate of vitamin D deficiency in different gender groups in 168 participants (male, 101 and female, 67) both of the year 2022 and 2023, *p < 0.05.

Among the 168 individuals who underwent 25(OH)D tests in both years, the prevalence of vitamin D deficiency in 2022 was 58.3%, higher than the 47.0% in 2023, with a statistically significant difference (*p* = 0.038). Among males, the prevalence of deficiency in 2022 was 54.5%, higher than the 40.6% in 2023, with a statistically significant difference (*p* = 0.049). No statistically significant difference was observed in the prevalence of vitamin D deficiency among females, as presented in Figure 3.

## 4 Discussion

The main findings of this study demonstrate a significant increase in overall 25-hydroxyvitamin D (25(OH)D) levels from 2022 to 2023, particularly notable in males. The prevalence of vitamin D deficiency also showed a significant decrease during this period. These findings can be attributed to the significant reduction in outdoor activities and sunlight exposure during the COVID-19 lockdown periods in 2022. Henan Province experiences substantial seasonal variations in sunlight, crucial for vitamin D synthesis. The lockdown measures enforced during the pandemic, particularly from January to February, May, and October to December 2022, coincided with periods of lower sunlight exposure, exacerbating vitamin D deficiency. Additionally, the heightened awareness of health and possible increased use of vitamin D supplements post-pandemic may have contributed to the improved vitamin D status observed in 2023.

Following the onset of the COVID-19 pandemic, China implemented stringent control strategies aimed at effectively mitigating disease transmission. These measures, however, imposed significant restrictions on individuals’ mobility, with some even being confined to their residences during the peak of the outbreak. Throughout 2022, the city of Zhengzhou experienced a total of 107 days characterized by rigorous control measures, resulting in a marked reduction in outdoor activities among the populace. It was not until December 14, 2022, that these stringent control policies were abruptly lifted, leading to a rapid propagation of the virus. By January 6, 2023, the Health Commission of Henan Province reported an infection rate of 89% for COVID-19 across the province, with urban areas exhibiting a rate of 89.1% and rural areas at 88.9%. Subsequently, the daily lives of individuals returned to a state of normalcy prior to the pandemic. The principal source of vitamin D synthesis in the human body stems from direct cutaneous exposure to sunlight, particularly ultraviolet B radiation (2). Consequently, the protracted duration of containment measures in Zhengzhou, Henan during 2022 is anticipated to exert an influence on the population’s vitamin D levels and the prevalence of vitamin D insufficiency.

Vitamin D plays a crucial role in the absorption of calcium which exerts pivotal roles in diverse physiological processes encompassing cellular signaling, hemostasis, myocyte contractility, and neural modulation (21). Moreover, investigations have elucidated the ability of vitamin D to facilitate phosphorus absorption, thereby directly influencing the calcium-phosphorus balance and skeletal metabolism in the human system (22). Epidemiological evidence has firmly established associations linking vitamin D deficiency with an array of chronic ailments, including autoimmune disorders such as multiple sclerosis, type 1 diabetes, and rheumatoid arthritis, as well as cardiovascular disease, type 2 diabetes, neurocognitive impairments, and infectious diseases, notably including COVID-19 (23–26).

Henan Province is situated in the central-eastern region of China, primarily characterized by expansive plains and encompassing an area of 16.7 square kilometers. Its geographic center is located at coordinates 33.5°N and 113.3°E. The province primarily falls within the warm temperate zone, with the southern part transitioning from subtropical to warm temperate, featuring a continental monsoon climate with well-defined seasonal variations. The four seasons are delineated as follows: Winter (December to February), Spring (March to May), Summer (June to August), and Autumn (September to November). The annual average temperature in Zhengzhou, the provincial capital, is approximately 15.6°C. August is the hottest month, with a mean temperature of 25.9°C, while January is the coldest, averaging 2.15°C. The region experiences a frost-free period of roughly 209 days and an annual sunshine duration of approximately 1869.7 h.

In this study, we observed a substantial decline in the number of individuals seeking medical examinations at our institution due to the COVID-19 pandemic, particularly during January and October to December of 2022, which exhibited a significant decrease compared to the corresponding periods in 2023. Concurrently, the pandemic of COVID-19 resulted in a decline in the overall serum 25(OH)D levels and an increase in the overall prevalence of vitamin D deficiency within the adult population. When stratifying by gender, we found that the impact of the COVID-19 pandemic was more pronounced among males than females. Specifically, in males, the serum 25(OH)D levels in 2023 were significantly higher than those in 2022, and the prevalence of vitamin D deficiency in 2023 was significantly lower than that in 2022. However, no significant differences in serum 25(OH)D levels and deficiency rates were observed between the 2 years among females. When comparing genders within the same year, the serum vitamin D levels in females were slightly higher than those in males in 2022, and the prevalence of vitamin D deficiency was lower than that in males, albeit these differences did not reach statistical significance. This phenomenon may be attributable to the fact that vitamin D is a fat-soluble vitamin stored in adipose tissue, and the disparity in body fat composition between genders may play a contributory role (27). In 2023, this pattern was reversed, with significantly higher serum vitamin D levels and significantly lower deficiency rates observed among males compared to females. This intriguing finding may be elucidated by the impact of the COVID-19 pandemic, as even though both males and females experienced reduced outdoor activity, females tend to adhere more diligently to sunscreen usage. Research has demonstrated that sunscreen with SPF30 can diminish vitamin D synthesis by 95% (28). Consequently, the impact of the COVID-19 pandemic appears to have a greater effect on males than females. This phenomenon was further corroborated by the analysis of vitamin D levels in 168 individuals examined in both 2022 and 2023. The mean serum 25(OH)D levels in 2023 were higher than those in 2022, both in the overall population and when comparing the same gender. Furthermore, the prevalence of vitamin D deficiency in 2023 was lower than that in 2022. Although disparities in 25(OH)D levels were noted among females between the 2 years, no significant differences in vitamin D deficiency rates were observed. In contrast, no disparities in 25(OH)D levels were observed among males between the 2 years, but a significant difference in vitamin D deficiency rates was evident. In the comparison of different age groups, it was observed that the vitamin D levels in the 40–49 age group were significantly lower in 2022 compared to 2023. Additionally, the prevalence of vitamin D deficiency in this age group was significantly higher in 2022 than in 2023. Moreover, irrespective of the year (2022 or 2023), there was a gradual increase in vitamin D levels and a decrease in deficiency rates with advancing age. These findings are consistent with previous research conducted in various regions of China and other countries globally (29, 30). The observed pattern can potentially be attributed to increased outdoor activity among older individuals after retirement, considering that the retirement age in China is 60 for males and 50 or 55 for females. Regarding the monthly comparison, it was noted that vitamin D levels in February, March, and April of 2022 were significantly lower than the corresponding months in 2023. Similarly, the deficiency rates of vitamin D in 2022 were higher compared to 2023 during the same months. These outcomes align with previous investigations in children conducted at Henan Children’s Hospital (20). Furthermore, this study revealed that vitamin D levels were highest during the summer months (June, July, August, and September) in both 2022 and 2023, followed by a gradual decline, reaching the lowest levels during the winter season. Overall, vitamin D levels during the summer and autumn seasons were significantly higher compared to the winter and spring seasons. These findings are consistent with prior studies conducted in other regions of China and other countries (30–32). The observed trends can be attributed to longer daylight hours, elevated ultraviolet radiation levels during the summer, as well as favorable weather conditions and increased outdoor activity time during the autumn season, all of which contribute to higher vitamin D levels.

This study represents the inaugural comparison of adult vitamin D levels and deficiency rates during and post the COVID-19 pandemic in Henan Province of China. Nonetheless, several limitations warrant consideration. Firstly, the study cohort primarily emanated from Henan Province, a plain region in China, thereby potentially engendering variances when juxtaposed with diverse locales. Secondly, the implementation of stringent containment measures compelled individuals to adhere to home confinement, consequently yielding a constrained sample size during this period. Notably, the study lacked comprehensive documentation pertaining to individual-specific outdoor activity duration, dietary habits, and vitamin D supplementation regimens, thereby potentially introducing inherent biases in the derived findings.

In conclusion, this study demonstrates that the COVID-19 pandemic significantly increased the proportion of vitamin D deficiency in the adult population, particularly in males. These findings underscore the importance of sufficient outdoor activities for maintaining adequate vitamin D levels.

## Data availability statement

The raw data supporting the conclusions of this article will be made available by the authors, without undue reservation.

## Ethics statement

The studies involving humans were approved by the Henan Provincial People’s Hospital Ethics Review Committee. The studies were conducted in accordance with the local legislation and institutional requirements. The ethics committee/institutional review board waived the requirement of written informed consent for participation from the participants or the participants’ legal guardians/next of kin because informed consent was waived due to the retrospective nature of the study.

## Author contributions

YC: Writing – original draft. GK: Writing – review & editing.
